# Characterization, Antibiotic Susceptibility, and Clonal Analysis of Carbapenem-Resistant Klebsiella pneumoniae From Different Clinical Cases

**DOI:** 10.7759/cureus.71889

**Published:** 2024-10-19

**Authors:** Zanan M Taha

**Affiliations:** 1 Pathology and Microbiology, University of Duhok, College of Veterinary Medicine, Duhok, IRQ

**Keywords:** antimicrobial resistance, antimicrobial stewardship, carbapenem-resistant klebsiella pneumoniae, clonal analysis, public health

## Abstract

Introduction: Carbapenem-resistant *Klebsiella pneumoniae *(*CRKP*) is recognized for its great ability to resist prescription drugs and its association with severe infections in humans.

Objectives: This study was designed to evaluate the characteristic resistance spectrum, to characterize the implicated carbapenem-resistant genes (CRGs), and to determine the extent of genetic diversity among *Klebsiella pneumoniae* isolates from human clinical cases in Duhok province.

Methodology: The VITEK-2 system was used to investigate the phenotypic antibiotic susceptibility of 23 *K. pneumoniae* isolated from distinct human clinical situations, multiplex PCR was used to assign the key common carbapenem-resistant genes (*IMP*, *OXA48*-like, *bla-NDM*, and *KPC*) in phenotypically carbapenem-resistant isolates, and the Enterobacterial Repetitive Intergenic Consensus Polymerase Chain Reaction (ERIC-PCR) assay was utilized to ascertain the clonal associations among those isolates.

Results: Phenotypic resistance analysis revealed high resistance rates to various antibiotics, with all isolates exhibiting multidrug resistance (MDR). Coronavirus disease 2019 (COVID-19) patient isolates demonstrated significantly higher resistance compared to other sources. In addition, all isolates showed complete phenotypic resistance to carbapenems, PCR screening for CRGs identified blaOXA-48 as the predominant gene, present in all isolates. Genetic fingerprinting revealed diverse genotypes, with COVID-19 patient isolates exhibiting high similarity, contrasting with maximum diversity in non-COVID-19 clinical isolates.

## Introduction

Carbapenem-resistant *Klebsiella pneumoniae *(*CRKP*) is a formidable pathogen, known for its high level of resistance to antibiotics and its association with severe infections in both human and animal populations. The emergence and rapid dissemination of *CRKP *pose significant challenges to public health worldwide, as these bacteria are often resistant to multiple antibiotics [1]. *Klebsiella pneumoniae* is among the most serious multidrug-resistant pathogens, alongside *Acinetobacter baumannii*, *Enterococcus faecium*, *Staphylococcus aureus*, *Pseudomonas aeruginosa*, and *Enterobacter* species, collectively referred to as the "ESKAPE" group, which are major contributors to hospital-acquired infections. This bacterium can endure in hospital environments and remain viable for extended periods on dry surfaces [2].

The increasing prevalence of *CRKP *in clinical settings has been well documented. These pathogens are frequently implicated in hospital-acquired infections, including pneumonia, bloodstream infections, and urinary tract infections, particularly among patients with compromised immune systems [3,4].

To treat *K. pneumoniae* infections, antimicrobial medicines have been extensively utilized. However, unregulated utilization of such medications and the repeated exposure of different *K. pneumoniae* isolates to antibacterial agents result in characteristics that are resistant to multiple drugs [5]. The resistance mechanisms in *CRKP *are often mediated by carbapenemase enzymes, such as KPC, NDM, OXA-48, and VIM, which hydrolyze carbapenems and other β-lactam antibiotics, rendering them ineffective [6]. When treating infections caused by bacteria that are resistant to multiple drugs, carbapenems are sometimes thought of as the final resort. They are crucial for treating severe infections caused by Gram-negative bacteria, including *K. pneumoniae* [7].

Research efforts have focused on understanding the molecular epidemiology of *CRKP*, including clonal analysis and genomic sequencing, to track the spread of these resistant strains and implement appropriate infection control measures [8]. To understand the complex epidemiology and transmission dynamics of this bacterium, researchers and healthcare professionals have turned to clonal analysis, a powerful tool that delves into the genetic relatedness and diversity of *CRKP *strains [9]. The objectives of this study were therefore to determine the antimicrobial resistance profiles of *K. pneumoniae* isolates from human sources, to examine the existence of key resistance genes in isolates that are phenotypically resistant to carbapenems and finally, and to characterize the genetic diversity and clonal relationships among these isolates using molecular typing methods (Enterobacterial Repetitive Intergenic Consensus Polymerase Chain Reaction (ERIC-PCR)).

## Materials and methods

Bacterial strains

Clinical *K. pneumoniae* isolates were collected from Duhok Research Centre (DRC) (College of Veterinary Medicine, University of Duhok), Duhok, Iraq, from August 2021 until July 2022. A total of 23 isolates from various clinical conditions were collected, including urine from urinary tract infection (n = 3), blood culture from bacteremia cases (n = 2), sputum from respiratory tract infection (n = 3) and non-coronavirus disease 2019 (COVID-19), pus from wounds (n = 3), and an additional 12 isolates from a pharyngeal swab in cases of severe pneumonia in COVID-19-suffering patients. Samples’ information, including the sample type and source and the patient's gender, was extracted from laboratories’ information systems. Then, isolates were cultured on MacConkey agar (LabM, UK) and confirmed as *K. pneumoniae* through their morphological characteristics (large mucoid colonies with pink coloration) [10], Vitek2 automated system (BioMerieux, Marcyl'Étoile, France) for Gram-negative bacteria, and PCR amplification of the gyrA gene.

DNA extraction

The standard protocol for DNA extraction followed the procedure of Taha and Yassin [11], with slight modifications. Five to six pure colonies with similar morphology were selected from MacConkey agar and combined with 500 µL of sterile double-distilled water in a 1.5 ml tube. The mixture was vortexed for at least 15 seconds and then heated at 95°C for 12 minutes. Afterward, the samples were rapidly cooled on ice, and the cooled suspension was centrifuged. Three hundred fifty µL of the supernatant was used as the DNA template for PCR. The purity and concentration of the extracted DNA were assessed using a nanodrop (Thermo Scientific, USA).

PCR detection of *K. pneumoniae*

 A 441-bp PCR fragment of the gyrAgene (F-5′- CGCGTACTATACGCCATGAACGTA-3′) and (R-5′- ACCGTTGATCACTTCGGTCAGG-3′) were used to confirm all tested strains. The PCR conditions were followed by Fatima et al. [12].

Susceptibility testing to “antibiotics”

Antibiotic susceptibility testing was carried out using the Vitek2 automated system with the VITEK 2 AST-N335 cards (BioMerieux, France). A panel of 28 antibiotics, including amoxicillin (AMX), amoxicillin/clavulanic acid (AMC), piperacillin-tazobactam (PIP-TAZ), cefuroxime (CXM), cefuroxime Axetil (CXM-AX), cefoxitin (CFX), cefixime (CFM), ceftazidime (CAZ), fosfomycin (FOS), ceftriaxone (CRO), cefepime (CEF), cefotaxime (CTX), ertapenem (ETP), imipenem (IPM), meropenem (MEM), amikacin (AMK), gentamicin (GEN), ciprofloxacin (CIP), tigecycline (TGC), cephalothin (CF), tobramycin (TOB), nitrofurantoin (NIT), colistin (COL), netilmicin (NET), levofloxacin (LVX), tetracycline (TET), erythromycin (ERY), and trimethoprim-sulfamethoxazole (SXT) were tested. *CRKP *were defined as the strains that are resistant to one of the three carbapenems (imipenem, ertapenem, or meropenem) [13]. Any isolate that exhibited resistance to three or more drugs was classified as having multiple resistance to antibiotics.

Detection of carbapenem resistance genes

PCR was used to detect the most frequent carbapenemase genes (bla KPC, bla IMP, bla NDM, and blaOXA-48-like) [14] (Table 1). Multiplex PCR was performed in a total volume of 20 µl containing 10 µl of Hot-Start Premix (Genet Bio, Korea), 0.25 pmol/µl each of the forward and reverse primers, and 1 µl of template DNA (100-150 ng/µl). The remaining 20 µl was completed with 7 µl of sterile distilled water. The PCR program was as follows: DNA was initially denaturated at 95 °C for three minutes, followed by 30 cycles of denaturation at 95 °C for 30 seconds, primer annealing at 56 °C for 30 seconds, extension at 72 °C for 45 seconds, and an ending extension at 72 °C for five minutes.

**Table 1:** Specific primers were used for the detection of carbapenem-resistance genes

Gene name	Sequence (5’–3’)	Product size (bp)
IMP-F IMP-R	F-GGAATAGAGTGGCTTAAYTCTC R-GGTTTAAYAAAACAACCACC	232
OXA48-like-F OXA48-like-R	F-GCGTGGTTAAGGATGAACAC R-CATCAAGTTCAACCCAACCG	438
blaNDM -F blaNDM -R	F-GGTTTGGCGATCTGGTTTTC R-CGGAATGGCTCATCACGATC	621
KPC-F KPC-R	F-CGTCTAGTTCTGCTGTCTTG R-CTTGTCATCCTTGTTAGGCG	798

ERIC PCR and diversity analysis

All 23 strains were analyzed for DNA fingerprinting and diversity analysis using ERIC PCR, amplifying the ERIC primer sequences (ERIC1: 5'-ATGTAAGCTCCTGGGGATTCAC-3' and ERIC2: 5'-AAGTAAGTGACTGGGGTGAGCG-3') previously described by Mare et al. [15]. The PCR reaction settings were followed by Alfeky et al. [16]. All PCR products were electrophoresed using a 1.5% agarose gel prepared in 1X Tris-acetate EDTA (TAE) buffer and stained with a red-safe DNA staining solution (GeNetBio, Korea).

Evaluation of ERIC PCR results

An image with 23 wells representing all isolates of *K. pneumoniae* were analyzed using the GelJ software version 2.0 (available at https://sourceforge.net/projects/gelj/) to generate a dendrogram [17]. The isolates were clustered using the unweighted pair group method with an arithmetic mean (UPGMA) and a dice similarity coefficient with a tolerance of around 1%. Isolates with a similarity coefficient equal to or above 80% (similarity criterion of ≥80%) were classified as having an identical genotype [18]. The clustering of the isolates depended on the different sample sources and genders.

Statistical analysis

ANOVA, or one-way analysis of variance, was used to assess all the data [19]. Using IBM SPSS Statistics for Windows, Version 20.0 (released 2011, IBM Corp., Armonk, NY), the Duncan multiple-range test was used to identify specific differences between the groups. P≤0.05 was the acceptable limit of significance.

Ethical consideration

An assigned permission document with reference no. 978 (date: October 13, 2024) was gotten from DRC, allowing the author to use the bacterial isolates for research purposes.

## Results

Phenotypic-resistant profile

The rate of phenotypic resistance profile for all isolates was as follows: all isolates (100%) were found resistant to each of amoxicillin, amoxicillin/clavulanic acid, piperacillin-tazobactam, cefuroxime, cefuroxime axetil, cefoxitin, cefixime, ceftazidime, ceftriaxone, cefepime, cefotaxime, ertapenem, imipenem, meropenem, tobramycin, cephalothin, netilmicin and erythromycin, (96.4% to tetracycline; 91.3% to each of nitrofurantoin, trimethoprim-sulfamethoxazole, and ciprofloxacin; 78.2% to each of levofloxacin and fosfomycin; 61% to amikacin; 52.1% to colistin; and 39.1% to gentamicin). None of the isolates were resistant to tigecycline. All isolates were 100% MDR (Table 2).

**Table 2:** Antibiotic-resistant profile of 23 K. pneumoniae isolated from different clinical samples AMX: amoxicillin, AMC: amoxicillin/clavulanic acid, PIP-TAZ: piperacillin-tazobactam, CXM: cefuroxime, CXM-AX: cefuroxime Axetil, CFX: cefoxitin, CFM: cefixime, CAZ: ceftazidime, CRO: ceftriaxone, CEF: cefepime, CTX: cefotaxime, ETP: ertapenem, IPM: imipenem, MEM: meropenem, TOB: tobramycin, CF: cephalothin, NET: netilmicin, ERY: erythromycin, FOS: fosfomycin, AMK: amikacin, GEN: gentamicin, CIP: ciprofloxacin, NIT: nitrofurantoin, COL: colistin, LVX: levofloxacin, TET: tetracycline, SXT: trimethoprim-sulfamethoxazole, TGC: tigecycline. ●: statistically significant (ANOVA with the Duncan multiple range test was used to determine the p value); MDR: multi-drug resistant

Antibiotics/sample source	Bacteremia no. (n = 2)	Wound no. (n = 3)	UTI no. (n = 3)	Non-COVID-19 pneumonia no. (n = 3)	COVID-19 no. (n = 12) ● (p < 0.05)	Total resistant no. (%) (n = 23)
AMX	2	3	3	3	12	23 (100)
AMC	2	3	3	3	12	23 (100)
PIP-TAZ	2	3	3	3	12	23 (100)
CXM	2	3	3	3	12	23 (100)
CXM-AX	2	3	3	3	12	23 (100)
CFX	2	3	3	3	12	23 (100)
CFM	2	3	3	3	12	23 (100)
CAZ	2	3	3	3	12	23 (100)
CRO	2	3	3	3	12	23 (100)
CEF	2	3	3	3	12	23 (100)
CTX	2	3	3	3	12	23 (100)
ETP	2	3	3	3	12	23 (100)
IPM	2	3	3	3	12	23 (100)
MEM	2	3	3	3	12	23 (100)
TOB	2	3	3	3	12	23 (100)
CF	2	3	3	3	12	23 (100)
NET	2	3	3	3	12	23 (100)
ERY	2	3	3	3	12	23 (100)
TET	2	2	3	3	12	22 (96.4)
NIT	0	3	3	3	12	21 (91.3)
SXT	0	3	3	3	12	21 (91.3)
CIP	0	3	3	3	12	21 (91.3)
LVX	1	2	3	3	9	18 (78.2)
FOS	1	3	2	0	12	18 (78.2)
AMK	2	1	1	3	7	14 (61)
COL	0	0	0	0	12	12 (52.1)
GEN	0	3	3	3	0	9 (39.1)
TGC	0	0	0	0	0	0
Total resistant pattern	42 (7.4)	74 (13)	75 (13.2)	75 (13.2)	304 (53.3)	570 (100)
MDR	2 (100)	3 (100)	3 (100)	3 (100)	12 (100)	23 (100)

Regarding the sample source, isolates from COVID-19 showed a higher resistant rate (53.3%; p < 0.05) when compared with the other sources, followed by 13.2% for each of the UTI and non-COVID-19 pneumonias, 13% in cases of wound infection, and the lowest rate of resistance was seen in bacteremia (7.4%) (Table 2).

Concerning gender, the highest resistant rate was observed in male isolates (56.3%) than in female isolates (43.4%) (p > 0.05). Gender variations in the resistance rate were not statistically significant, with the exception of amikacin, where isolates from females were shown to be considerably resistant (p ≤ 0.05) (Table 3).

**Table 3:** Comparison of antibiotic resistant patten between male and female AMX: amoxicillin, AMC: amoxicillin/clavulanic acid, PIP-TAZ: piperacillin-tazobactam, CXM: cefuroxime, CXM-AX: cefuroxime Axetil, CFX: cefoxitin, CFM: cefixime, CAZ: ceftazidime, CRO: ceftriaxone, CEF: cefepime, CTX: cefotaxime, ETP: ertapenem, IPM: imipenem, MEM: meropenem, TOB: tobramycin, CF: cephalothin, NET: netilmicin, ERY: erythromycin, FOS: fosfomycin, AMK: amikacin, GEN: gentamicin, CIP: ciprofloxacin, NIT: nitrofurantoin, COL: colistin, LVX: levofloxacin, TET: tetracycline, SXT: trimethoprim-sulfamethoxazole, TGC: tigecycline. ●: statistically significant; ▼: statistically non-significant (ANOVA with the Duncan multiple range test was used to determine the p value); MDR: multi-drug resistant

Antibiotics/sample source	Total resistant isolates no. (n = 23)	Male (n =13)	Female (n =10)
AMX	23	13	10
AMC	23	13	10
PIP-TAZ	23	13	10
CXM	23	13	10
CXM-AX	23	13	10
CFX	23	13	10
CFM	23	13	10
CAZ	23	13	10
CRO	23	13	10
CEF	23	13	10
CTX	23	13	10
ETP	23	13	10
IPM	23	13	10
MEM	23	13	10
TOB	23	13	10
CF	23	13	10
NET	23	13	10
ERY	23	13	10
FOS	18	11	7
AMK	14	6	8● (p < 0.05)
GEN	9	6	3
CIP	21	12	9
NIT	21	12	9
COL	12	6	6
LVX	18	9	9
TET	22	13	9
SXT	21	12	9
TGC	0	0	0
Total resistant pattern	570 (100)	321 (56.3) ▼	249 (43.4) ▼

Carbapenem-resistant genes in phenotypic carbapenem-resistant strains

 The study results displayed a complete phenotypic resistance of human *K. pneumoniae* isolates to carbapenems (all 23 human strains were carbapenem-resistant). A PCR assay was used for screening of the four most common CRGs (IMP, OXA48-like, blaNDM, and KPC) in phenotypically carbapenem-resistant isolates. The results showed that all isolates (100%) carried at least one CRG. Out of the 23 CRG-positive isolates, blaOXA-48 was identified in all 23 (100%) isolates, while bla-NDM was only found in six (26%) isolates. In addition, the existence of both bla-NDM and blaOXA-48 together was detected in six (26%) isolates. None of the isolates tested positive for both IMP and KPC (Table 4). 

**Table 4:** Distribution of carbapenem resistant genes (CRGs) among human patients by the sample source

CRGs	Bacteremia (n = 2)	Wound (n = 3)	Non-COVID-19 pneumonia (n = 3)	UTI (n = 3)	COVID-19 (n = 12)	Total (n = 23)
OXA48-like	2	3	3	3	12	23 (100)
blaNDM	2	-	3	1	0	6 (26)
OXA48-like + blaNDM together	2	-	3	1	0	6 (26)
IMP	-	-	-	-	-	-
KPC	-	-	-	-	-	-

DNA fingerprinting and diversity analysis

**Figure 1:**
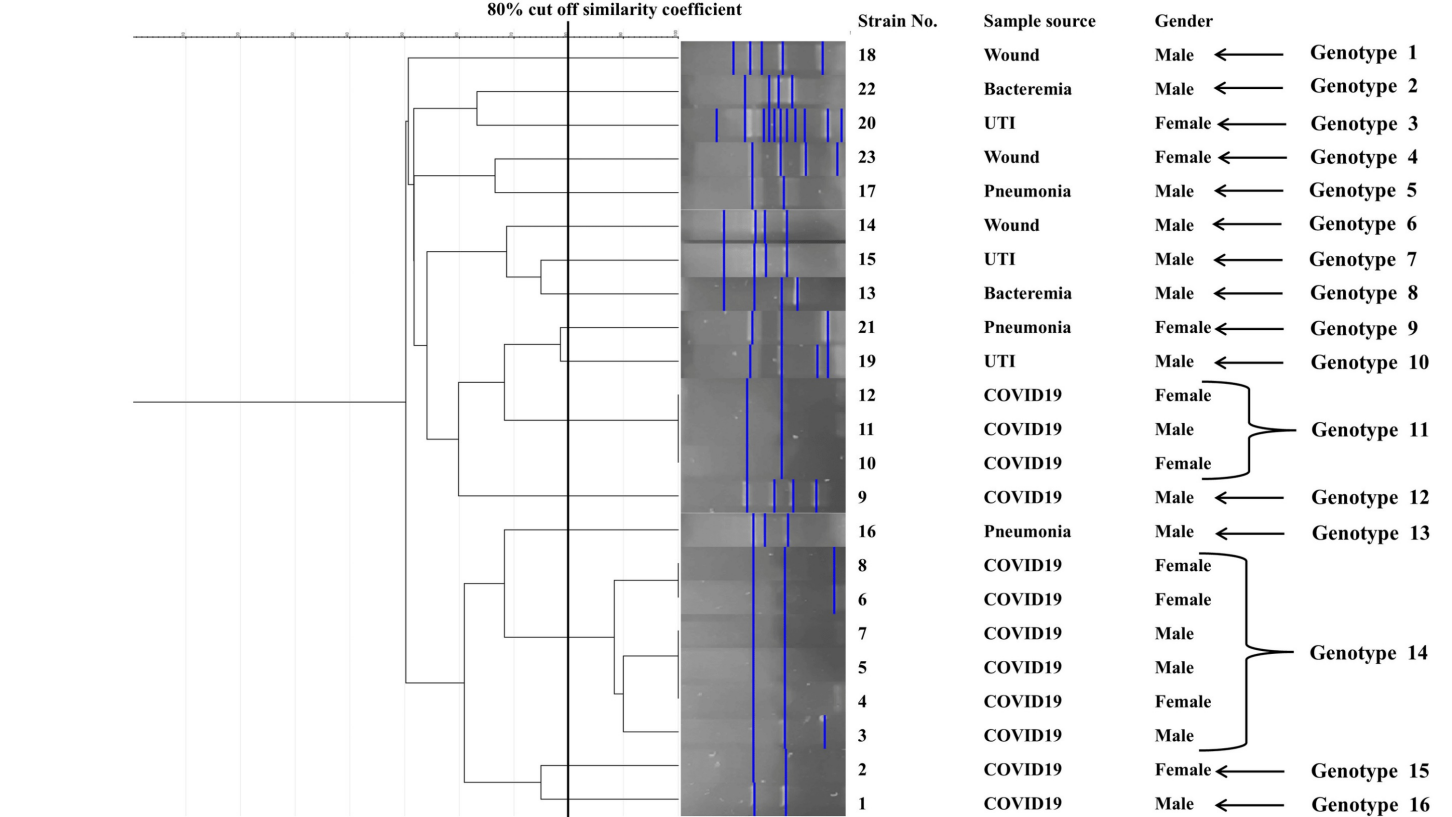
Dendrogram generated from ERIC-PCR showing the banding pattern of 23 K. pneumoniae strains isolated from different clinical conditions

The results showed that the genetic similarity among 23 *K. pneumoniae* isolates was between 51% and 100%, and all isolates were grouped into 16 genotypes (1-16) according to the 80% cut-off similarity coefficient, including two common genotypes and 14 unique types. The majority of clones were of genotypes 11 and 14, comprising 9/23, or 39% of all isolates, and all of them were from COVID-19 patients. Genotype 14 contained six strains (three males and three females), while genotype 11 contained three strains (one male and two females). The remainder of the genotypes consisted of just one strain (Figure 1).

It is remarkable to observe that isolates from COVID-19 patients, irrespective of gender, shared the majority of genetic characteristics., while the maximum genetic diversity was seen in other sample sources (non-COVID-19 clinical). All strains from non-COVID-19 clinical samples were clustered with a single genotype, and there was no evidence of cross-transmission of infection (Figure 1). 

## Discussion

Determining the antimicrobial resistance profiles of *CRKP* is crucial for understanding the extent of resistance and developing effective treatment protocols, which in turn aid in creating targeted antibiotic stewardship programs and clinical decisions to reduce misuse [20]. In addition, studying the clonal analysis of this pathogen in multiple clinical settings is intended to provide critical insights into the epidemiology and transmission dynamics. This might enable public health officials to identify and control outbreaks more effectively, preventing the spread of *CRKP *within human populations [21].

Phenotypic resistant profile

The study presents a comprehensive phenotypic resistance profile of *K. pneumonia*e isolates from clinical human sources, revealing a high prevalence of MDR, particularly in COVID-19 patients, who exhibited the highest resistance rates compared to those from other sources. All isolates were resistant to most routinely prescribed antibiotics. This extensive resistance pattern is consistent with global reports of increasing antimicrobial resistance in *K. pneumoniae*, complicating treatment strategies [22]. The significant resistance to β-lactam antibiotics, including amoxicillin, amoxicillin/clavulanic acid, and piperacillin-tazobactam, was seen in this investigation. Similar resistance rates were observed for various cephalosporins, which are critical for treating Gram-negative bacterial infections [23].

These drugs are often used as second-line treatments for MDR bacterial infections; thus, their reduced efficacy poses a significant therapeutic challenge [24]. These resistances to the above antibiotics could be attributed to several conditions, including prior exposure [25], prolonged hospitalization (particularly in COVID-19 hospitals), in which extended stays in the hospital, especially in the ICU, increase exposure to resistant bacteria [26], immunosuppression, in which patients with compromised immune status make it harder to clear infections, promoting the persistence and spread of resistant strains [27], and cross-transmission in healthcare settings [28].

Notably, carbapenem resistance was also high, with all human isolates showing resistance to ertapenem, imipenem, and meropenem. This is a serious problem since, as the last option, this kind of antibiotic is administered to treat infections brought on by “Gram-negative bacteria” that are resistant to most drugs [29]. This may be due to the increased person's exposure to these medications, since imipenem or meropenem are the medications of choice for treating serious infections caused by bacteria around the globe, especially in COVID-19 cases [30].

Tigecycline and colistin were used as secondary options for the treatment of carbapenem resistance bacteria [31,32]. However, the absence of resistance to tigecycline among all isolates is encouraging, indicating its continued effectiveness against MDR *K. pneumoniae*. Nonetheless, the sole reliance on tigecycline should be approached with caution to prevent the emergence of resistance [33]. However, the colistin resistance in this investigation was substantial, particularly in COVID-19.

The ability of *K. pneumoniae* to form biofilms significantly enhances the transfer of horizontally transferable colistin-resistant genes between bacteria [34,35]. This mechanism plays a vital role in disseminating antibiotic resistance genes, leading to the development of resistant strains on medical devices and tissues, which can result in challenging infections [36]. As a result, all isolates from COVID-19 were found to be colistin resistant (there was an outbreak of colistin resistance); this will suggest that the mechanism of colistin resistance may have arisen in these isolates by horizontal gene transfer. The use of colistin can select for plasmids carrying the mcr-1 gene, which confers resistance and can be transferred horizontally between different bacterial species, especially in the overcrowded status of COVID-19 hospitals, increasing the spread of resistance [37,38].

The study observed higher resistance rates in isolates from male patients compared to female patients, although this difference was not statistically significant (p˃0.05). These findings align with previous studies suggesting gender-related variations in antibiotic resistance, possibly due to differences in healthcare-seeking behavior and antibiotic usage patterns [39]. Men are generally less likely to seek medical care promptly, which can lead to more advanced infections by the time they receive treatment. This delay can result in the use of more aggressive antibiotic therapies, increasing the likelihood of developing antibiotic-resistant infections [40]. In addition, the observed significant resistance to amikacin in *K. pneumoniae* isolates from females compared to male isolates is explained by the selection resistance pressure brought on by the higher prior use of this medication to treat “urinary tract infections (UTIs)” [41].

Carbapenem-resistant genes in phenotypic carbapenem resistance

The ubiquitous presence of blaOXA-48 in all isolates underscores the dominant role of this gene in carbapenem resistance among clinical *K. pneumoniae *strains [42]. This finding aligns with global trends, where blaOXA-48 has been identified as a significant contributor to carbapenem resistance [43].

In addition, the co-occurrence of blaNDM and blaOXA-48 in some isolates further complicates treatment options, as these strains exhibit enhanced resistance profiles due to the presence of multiple resistance mechanisms. While the detection of blaNDM in some of them alone, without the co-occurrence of blaOXA-48, highlights the diverse genetic mechanisms contributing to resistance within this pathogen [44]. However, the absence of other CRGs suggests a possible geographical or epidemiological variability in the distribution of CRGs, as the predominance of specific resistance genes can vary by region and healthcare settings [45].

Genetic diversity and clonal analysis

The DNA fingerprinting and diversity analysis of *K. pneumoniae* isolates through ERIC-PCR fingerprinting revealed significant genetic diversity among the isolates. This will highlight the broad genetic diversity within the population studied. This variation suggests that multiple clones of this bacterium are circulating in our region, which might originate in various geographic spots [46]. This might suggest that there were a variety of sources for bacterial transmission to human’s patients. To better understand the sources and pathways of these clones, a comprehensive genomic surveillance program should be implemented. This involve regular sampling and whole-genome sequencing of *K. pneumoniae* isolates from different hospitals and communities. Such an approach can help trace the origin and spread of specific clones, enabling more effective infection control strategies [47].

Interestingly, the highest genetic similarity was observed among isolates from COVID-19 patients, regardless of gender. This may reflect the nosocomial transmission within hospital settings [48]. The shared environment and medical equipment increase the likelihood of transmission of similar bacterial strains among patients [49]. In addition, prolonged hospital stays often require long-term hospitalization, leading to prolonged exposure to hospital-acquired pathogens. This increases the risk of acquiring and spreading genetically similar *K. pneumoniae* strains [26]. 

However, the heterogenicity of the genetic profile in non-COVID-19 patients suggests that there was no such cross-bacterial transmission between patients (patients have attended the hospital from diverse geographic regions as bacterial genetic diversity varies significantly across different geographic regions) [50,51] and that multiple clones of a single species of this bacterium are circulating in this area, and this is potentially due to less stringent transmission selective pressures compared to those within the COVID-19 hospital environment [18,52]. 

Limitations

The study presents several limitations that may impact the validity and generalizability of its findings. One major limitation is the insufficient number of bacterial isolates, which restricts the ability to fully understand the diversity and prevalence of bacterial strains involved. In addition, the study lacks comprehensive data on the previous history of antibiotic prescriptions for each patient. This omission hinders the analysis of potential correlations between prior antibiotic use and the presence or resistance patterns of bacterial isolates. These limitations underscore the need for larger sample sizes and more detailed patient history to enhance the robustness of future research in this area.

## Conclusions

The study highlights the extensive phenotypic resistance of *K. pneumoniae* isolates from human clinical sources. COVID-19 patient isolates exhibited the highest resistance rates. Male isolates showed higher resistance except for amikacin, which was higher in female isolates. All human isolates were completely resistant to carbapenems, carrying at least one carbapenem-resistant gene, predominantly blaOXA-48. Genetic analysis revealed significant diversity, with notable similarity among COVID-19 patient isolates and distinct genotypes from non-COVID-19 sources. These findings underscore the need for targeted antimicrobial stewardship and infection control strategies to manage resistant* K. pneumoniae*.

## References

[1] Hu Y, Ping Y, Li L, Xu H, Yan X, Dai H (2016). A retrospective study of risk factors for carbapenem-resistant Klebsiella pneumoniae acquisition among ICU patients. J Infect Dev Ctries.

[2] Ahmed MS, Abdulrahman ZF, Taha ZM (2024). The effect of silver nanoparticles on the antimicrobial activity of cloned nisin against extensively drug-resistant Acinetobacter baumannii". J Infect Public Health.

[3] Tzouvelekis LS, Markogiannakis A, Psichogiou M, Tassios PT, Daikos GL (2012). Carbapenemases in Klebsiella pneumoniae and other Enterobacteriaceae: an evolving crisis of global dimensions. Clin Microbiol Rev.

[4] Siddhardha B, Dyavaiah M, Syed A (2020). Model organisms for microbial pathogenesis, biofilm formation and antimicrobial drug discovery.

[5] Ibrahim ME (2023). Risk factors in acquiring multidrug-resistant Klebsiella pneumoniae infections in a hospital setting in Saudi Arabia. Sci Rep.

[6] Lai CC, Yu WL (2021). Klebsiella pneumoniae harboring carbapenemase genes in Taiwan: its evolution over 20 years, 1998-2019. Int J Antimicrob Agents.

[7] Mancuso G, De Gaetano S, Midiri A, Zummo S, Biondo C (2023). The challenge of overcoming antibiotic resistance in carbapenem-resistant gram-negative bacteria: "Attack on Titan". Microorganisms.

[8] Shen C, Lv T, Huang G, Zhang X, Zheng L, Chen Y (2023). Genomic insights into molecular characteristics and phylogenetic linkage between the cases of carbapenem-resistant Klebsiella pneumoniae from a non-tertiary hospital in china: a cohort study. Jundishapur J Microbiol.

[9] Zhang Y, Yang X, Liu C (2023). Increased clonal dissemination of OXA-232-producing ST15 Klebsiella pneumoniae in Zhejiang, China from 2018 to 2021. Infect Dis Poverty.

[10] Hartantyo SH, Chau ML, Koh TH (2020). Foodborne Klebsiella pneumoniae: Virulence potential, antibiotic resistance, and risks to food safety. J Food Prot.

[11] Taha Z, Yassin N (2019). Prevalence of diarrheagenic Escherichia coli in animal products in Duhok province, Iraq. Iran J Vet Res.

[12] Fatima S, Liaqat F, Akbar A (2021). Virulent and multidrug-resistant Klebsiella pneumoniae from clinical samples in Balochistan. Int Wound J.

[13] Potter RF, D'Souza AW, Dantas G (2016). The rapid spread of carbapenem-resistant Enterobacteriaceae. Drug Resist Updat.

[14] Hatrongjit R, Kerdsin A, Akeda Y, Hamada S (2018). Detection of plasmid-mediated colistin-resistant and carbapenem-resistant genes by multiplex PCR. MethodsX.

[15] Mare A, Man A, Ciurea CN, Pintea-Simon IA, Ianoși ES, Gîrbovan CE, Toma F (2022). Serogroups and genetic diversity of diarrheagenic strains of Escherichia coli: a retrospective study. J Infect Dev Ctries.

[16] Alfeky AE, Tawfick MM, Ashour MS, El-Moghazy AA (2022). High prevalence of multi-drug resistant methicillin-resistant Staphylococcus aureus in tertiary Egyptian hospitals. J Infect Dev Ctries.

[17] Heras J, Domínguez C, Mata E, Pascual V, Lozano C, Torres C, Zarazaga M (2015). GelJ--a tool for analyzing DNA fingerprint gel images. BMC Bioinformatics.

[18] Saleh Ahmed M, Abdulrahman ZF, Taha ZM (2023). Risk factors of clonally related, multi, and extensively drug-resistant Acinetobacter baumannii in severely ill COVID-19 patients. Can J Infect Dis Med Microbiol.

[19] Taha ZM, Mustafa SI, Ahmed CJ (2023). Multidrug-resistant and clonal dispersion of enterotoxigenic Escherichia coli from ready-to-eat meat products in Duhok province, Iraq. Iraqi J Vet Sci.

[20] Majumder MA, Rahman S, Cohall D, Bharatha A, Singh K, Haque M, Gittens-St Hilaire M (2020). Antimicrobial stewardship: fighting antimicrobial resistance and protecting global public health. Infect Drug Resist.

[21] Berglund B, Hoang NT, Lundberg L (2021). Clonal spread of carbapenem-resistant Klebsiella pneumoniae among patients at admission and discharge at a Vietnamese neonatal intensive care unit. Antimicrob Resist Infect Control.

[22] Martin RM, Bachman MA (2018). Colonization, infection, and the accessory genome of Klebsiella pneumoniae. Front Cell Infect Microbiol.

[23] Zhang R, Liu L, Zhou H (2017). Nationwide surveillance of clinical carbapenem-resistant Enterobacteriaceae (CRE) strains in China. EBioMedicine.

[24] Rodríguez-Baño J, Gutiérrez-Gutiérrez B, Machuca I, Pascual A (2018). Treatment of infections caused by extended-spectrum-beta-lactamase-, ampC-, and carbapenemase-producing enterobacteriaceae. Clin Microbiol Rev.

[25] Tumbarello M, Trecarichi EM, De Rosa FG (2015). Infections caused by KPC-producing Klebsiella pneumoniae: differences in therapy and mortality in a multicentre study. J Antimicrob Chemother.

[26] Peters L, Olson L, Khu DT (2019). Multiple antibiotic resistance as a risk factor for mortality and prolonged hospital stay: a cohort study among neonatal intensive care patients with hospital-acquired infections caused by gram-negative bacteria in Vietnam. PLoS One.

[27] Huemer M, Mairpady Shambat S, Brugger SD, Zinkernagel AS (2020). Antibiotic resistance and persistence-Implications for human health and treatment perspectives. EMBO Rep.

[28] Mitchell BG, Shaban RZ, MacBeth D, Wood CJ, Russo PL (2017). The burden of healthcare-associated infection in Australian hospitals: a systematic review of the literature. Infect Dis Health.

[29] Morris S, Cerceo E (2020). Trends, epidemiology, and management of multi-drug resistant gram-negative bacterial infections in the hospitalized setting. Antibiotics (Basel).

[30] Shin HS (2020). Empirical treatment and prevention of COVID-19. Infect Chemother.

[31] Zhang J, Yu L, Fu Y (2019). Tigecycline in combination with other antibiotics against clinical isolates of carbapenem-resistant Klebsiella pneumoniae in vitro. Ann Palliat Med.

[32] Zhang Y, Wang X, Wang S (2021). Emergence of colistin resistance in carbapenem-resistant hypervirulent Klebsiella pneumoniae under the pressure of tigecycline. Front Microbiol.

[33] Wang G, Zhao G, Chao X, Xie L, Wang H (2020). The characteristic of virulence, biofilm and antibiotic resistance of Klebsiella pneumoniae. Int J Environ Res Public Health.

[34] Nirwati H, Sinanjung K, Fahrunissa F (2019). Biofilm formation and antibiotic resistance of Klebsiella pneumoniae isolated from clinical samples in a tertiary care hospital, Klaten, Indonesia. BMC Proc.

[35] Olaitan AO, Morand S, Rolain JM (2014). Mechanisms of polymyxin resistance: acquired and intrinsic resistance in bacteria. Front Microbiol.

[36] Li L, Gao X, Li M (2024). Relationship between biofilm formation and antibiotic resistance of Klebsiella pneumoniae and updates on antibiofilm therapeutic strategies. Front Cell Infect Microbiol.

[37] Li R, Xie M, Zhang J (2017). Genetic characterization of mcr-1-bearing plasmids to depict molecular mechanisms underlying dissemination of the colistin resistance determinant. J Antimicrob Chemother.

[38] Shabban M, Fahim NAE, Montasser K, Abo El Magd NM (2020). Resistance to colistin mediated by mcr-1 among multidrug resistant gram negative pathogens at a tertiary care hospital, Egypt. J Pure Appl Microbiol.

[39] Schröder W, Sommer H, Gladstone BP, Foschi F, Hellman J, Evengard B, Tacconelli E (2016). Gender differences in antibiotic prescribing in the community: a systematic review and meta-analysis. J Antimicrob Chemother.

[40] Dias SP, Brouwer MC, van de Beek D (2022). Sex and gender differences in bacterial infections. Infect Immun.

[41] Cho SY, Choi SM, Park SH, Lee DG, Choi JH, Yoo JH (2016). Amikacin therapy for urinary tract infections caused by extended-spectrum β-lactamase-producing Escherichia coli. Korean J Intern Med.

[42] Wang CH, Ma L, Huang LY, Yeh KM, Lin JC, Siu LK, Chang FY (2021). Molecular epidemiology and resistance patterns of bla(OXA-48)Klebsiella pneumoniae and Escherichia coli: a nationwide multicenter study in Taiwan. J Microbiol Immunol Infect.

[43] Chen Y, Fang L, Yang Y (2021). Emergence of carbapenem-resistant Klebsiella pneumoniae harbouring bla (OXA-48)-like genes in China. J Med Microbiol.

[44] Kumarasamy KK, Toleman MA, Walsh TR (2010). Emergence of a new antibiotic resistance mechanism in India, Pakistan, and the UK: a molecular, biological, and epidemiological study. Lancet Infect Dis.

[45] Cai W, Kang J, Ma Y, Yin D, Song Y, Liu Y, Duan J (2023). Molecular epidemiology of carbapenem resistant Klebsiella pneumoniae in northern china: clinical characteristics, antimicrobial resistance, virulence and geographic distribution. Infect Drug Resist.

[46] De Koster S, Rodriguez Ruiz JP, Rajakani SG, Lammens C, Glupczynski Y, Goossens H, Xavier BB (2022). Diversity in the characteristics of Klebsiella pneumoniae ST101 of human, environmental, and animal origin. Front Microbiol.

[47] Arcari G, Carattoli A (2023). Global spread and evolutionary convergence of multidrug-resistant and hypervirulent Klebsiella pneumoniae high-risk clones. Pathog Glob Health.

[48] Xia J, Gao J, Tang W (2016). Nosocomial infection and its molecular mechanisms of antibiotic resistance. Biosci Trends.

[49] Tajeddin E, Rashidan M, Razaghi M (2016). The role of the intensive care unit environment and health-care workers in the transmission of bacteria associated with hospital acquired infections. J Infect Public Health.

[50] Truong DT, Tett A, Pasolli E, Huttenhower C, Segata N (2017). Microbial strain-level population structure and genetic diversity from metagenomes. Genome Res.

[51] Centeno CM, Legendre P, Beltrán Y, Alcántara-Hernández RJ, Lidström UE, Ashby MN, Falcón LI (2012). Microbialite genetic diversity and composition relate to environmental variables. FEMS Microbiol Ecol.

[52] Jernigan JA, Hatfield KM, Wolford H (2020). Multidrug-resistant bacterial infections in U.S. hospitalized patients, 2012-2017. N Engl J Med.

